# Knowledge implementation in health care management: a qualitative study

**DOI:** 10.1186/s12913-020-5043-8

**Published:** 2020-03-06

**Authors:** G. Roohi, G. Mahmoodi, H. Khoddam

**Affiliations:** 1 Department of Health Services Management, Islamic Azad University- Sari Branch, Sari, Iran; 2 Hospital Administration Research Center, Sari Branch, Sari, Iran; 3 Nursing Research Center, Golestan University of Medical Sciences, Gorgan, Iran

**Keywords:** Research implementation, PARIHS framework, Healthcare management system, Iran, Content analysis

## Abstract

**Background:**

The gap between knowledge and practice is a global issue, which increases wasteful spending in healthcare. There are several models and frameworks to address this gap and try to solve the challenge. Promoting Action on Research Implementation in Health Services (PARIHS) framework highlights the interaction of three main elements: evidence, context and facilitation, to implement research into practice, successfully. This framework can use as a tool to evaluate the situation and guide the changing. This study conducted to explain the status of knowledge implementation in Iran’s healthcare management system.

**Methods:**

This qualitative study was done by using a directive content analysis approach through conducting in-depth, structured interviews with 15 health managers based on the PARIHS framework. Guiding questions were based on the three main elements of the framework: evidence, context and facilitation. The content of the interviews entered into the Qualitative Data Analysis software (MAXQDA version 10) and, then, analyzed.

**Results:**

The most common source of evidence used by managers for decision-making was local information and previous experience. Evaluation more emphasized compared to other sub-elements of context, i.e. culture and leadership. In terms of facilitation, performing tasks by others was the dominant opinion.

**Conclusion:**

Our results showed that managers in the healthcare system of Iran use their own and other manager’s experience and the local information for decision-making and have no ideas about facilitation.

## Background

Nowadays, improving the quality of treatment and achieving the highest standard of care is one of the key goals of any healthcare system [1]. Recent rapid advances in medical sciences and technology have not only raised public awareness and improved economic status, but also increased people’s expectations of health services [2, 3]. Despite the point that millions of dollars are spent every year on healthcare, quality of health services is still poor and unfavorable [4].

Knowledge is an important source of wisdom and efficient actions in any organization [5]. Lack of synergy between knowledge and performance of health system is indeed a gap between knowledge and practice [6, 7]. Promotion of public health requires attention to a knowledge- and evidence-based decision making system [8] and human development is possible by storing, using and sharing knowledge [9].

Decision making in the health system has a significant relationship with the research findings and knowledge available in this field [10]. Until a common understanding between owners of knowledge and the health system is developed, the research findings are merely the dissemination of results and will not be effective for the patients and health system [11]. Lack of using knowledge in the health management system will waste financial resources, time and energy [12] and increase the costs imposed on the patients. It may be even detrimental to patients [13]. In addition, it will hinder implementation of the recommended healthcare services in terms of prevention, treatment and management [10, 11, 14].

Implementing knowledge into practice is a dynamic and interactive process that includes production, dissemination, exchange and application of knowledge to improve service delivery [15]. Barriers to knowledge utilization include lack of or inability to access knowledge resources, indifference towards the knowledge gained through research findings [16] and lack of time to find evidence that can help managers and policy makers [17]. Moreover, managers generally make decisions based on the information gained through recommendations and results of routine organizational measurements rather than research findings [18]. In recent decades, several models have been proposed on how to apply knowledge in practice or facilitate this process [19, 20]. The “Promoting Action on Research Implementation in Health Services” (PARIHS) is a conceptual framework that developed by Kitson et al. (1998) with the aim of promoting research implementation in practice. This model highlights the interaction of evidence, context and facilitation for successful implementation. These elements made up of some sub-elements, which define them. Evidence is defined by sub-elements of research findings, experiences of service providers and recipients as well as local information (i.e. Organizational knowledge); culture refers to the sub-elements of leadership and evaluation methods and the last element, facilitation, is defined by goals, roles and skills of individuals in/out of the organization that helps others make things easier [21]. Based on the framework, each sub-element is placed on a continuum from low to high (Table 1) [22] and can be used as a tool to evaluate and describe the current status of an organization in terms of research implementation in practice [23]. Considering the differences between various disciplines and organizations and the shortcomings in the implementation of management knowledge in the health system of Iran, we are aiming to use the above-mentioned framework to demonstrate the position of this discipline among other members of the healthcare service providers in terms of translating knowledge into practice.

**Table 1:** Elements of Promoting Action on Research Implementation in Health Systems (PARIHS) framework

Elements		sub elements	
		Low	High
Evidence	Research	• Poorly conceived, designed, and/or executed research • Seen as only type of evidence • Not valued as evidence • Seen as certain	• Well-conceived, deigned, and executed research, appropriate to the research question • Seen as one part of a decision • Valued as evidence • Lack of certain acknowledged • Judged as relevant • Importance weighted • Conclusion drown
	Clinical experience	• Anecdotal, with critical reflection and judgment • Lack of consensus within similar groups • Not valued as evidence • Seen as only type of evidence	• Clinical experience and expertise reflected upon, tested by individuals and groups • Consensus within similar groups • Valued as evidence • Seen as only type of evidence • Judged as relevant • Importance weighted • Conclusion drown
	Patient experience	• Not valued as evidence • Seen as only type of evidence • Patient not involved	• Valued as evidence • Multiple biographic used • Partnership with healthcare professionals • Seen as only type of evidence • Judged as relevant • Importance weighted • Conclusion drown
	Local data/ information	• Not valued as evidence • Lack of systematic methods for collection and analysis • Not reflected upon • Not Conclusion drown	• Valued as evidence • Collected and analysis systematically rigorously • Evaluated and reflected upon • Conclusion drown
Context	Culture	• Unclear valued and beliefs • Low regard for individuals • Task driven organization • Lake of consistency • Resources not allocated • Well integrated with strategic goals	• Abel to define cultures in terms of prevailing values / beliefs • Values individual staff and clients • Promotes learning organization • Consistency of individuals role/experience to value relationship with others teamwork • Power and authority • Rewards /recognition • Resources-human, financial, equipment, allocated • Initiative fits with strategic goals and is a key practice/patient issue
	Leadership	• Traditional, command, and control leadership • Lack of role clarity • Lack of teamwork • Poor organizational structures • Autocratic decision-making processes • Didactic approaches to learning/teaching/managing	• Transformational leadership • Role clarity • Effective teamwork • Effective organizational structures • Democratic-inclusive decision-making processes • Enabling/empowering approach to teaching/learning/managing
	Evaluation	• Absence of any form of feedback • Narrow use of performance information sources • Evaluations rely on single rather than multiple methods	• Feedback on Individual Team System performance • Use of multiple sources of information on performance • Use of multiple methods Clinical Performance Economic Experience evaluations
Facilitation	Purpose	Task Doing for others • Episodic contact • Practical/technical help • Didactic, traditional approach to teaching • External agents • Low intensity—extensive coverage	Holistic Role Enabling others • Sustained partnership • Developmental • Adult learning approach to teaching • Internal/external agents • High intensity—limited coverage
	Skills and attributes	Task/doing for others • Project management skills • Technical skills • Marketing skills • Subject/technical/clinical credibility	Holistic/enabling others • Cocounseling • Critical reflection • Giving meaning • Flexibility of role • Realness/authenticity

## Methods

The approach followed in this qualitative study was directed content analysis. In doing so, we traced the steps described by Hsieh and Shannon [24] to conduct the interviews and analyse the emerged content. In fact, this deductive approach could help form the study categories and subcategories before starting the data gathering corresponding with three main elements of PARIHS framework, i.e. Evidence, context and facilitation.

This study conducted in Iran health care system. The healthcare system in Iran managed with a centralized policy. It means, the Ministry of Health and Medical Education (MOHME) is responsible for policy making and managing the whole health system and the executive processes accomplished by the medical and educational universities in each province. For this reason, the university’s deans do the selection and appointment of the middle and top-level managers of hospitals and health care canters. These managers can be a general physician or medical specialist with or without an education in health care management. Therefore, the interviews carried out on 15 executive and top-level health managers with enough experiences in health and treatment sectors of Medical Sciences Universities in 2018–2019 (Table 2). The inclusion criteria were having at least 5 years of management experience in the field of healthcare and willingness to participate in the study. After obtaining the informed consent, the participants interviewed individually in a semi-structured manner. Since, the study based on the PARiHS framework; we use the diagnostic and evaluative questions in terms of the main elements (Evidence, Context and facilitation) of this model as an interview guide [25]. The interviews began with open and general questions about their managerial experiences, manner of decision-making, resource allocation and system evaluation. Then, to obtain the rich and specified data, the interview was guided to take examples and detailed explanations related to the framework’s elements.

**Table 2:** Demographic characteristics of the participants

Subject	Work experience (years)	Gender	Expertise/Specialty	Level of education	Main experiences
1	30	Male	Laboratory sciences	Doctorate	Deputy of cultural and students affairs
2	20	Male	Medical and health services management	PhD	Health Services Manager
3	30	Male	Nursing	MSc	Director of education
4	22	Male	Pediatric neurology	Medical doctor-Fellowship	Hospital CEO and deputy of treatment
5	21	Male	Pharmacology	Doctorate	Deputy of food and drug
6	20	Male	Nutrition	PhD	Health Services Manager
7	30	Male	Anesthesiology	MSc	Head of college and Vice-chancellor for cultural affairs
8	20	Male	General practitioner	Medical doctor	Director of treatment monitoring
9	21	Male	Reproductive health	PhD	Health center manager
10	29	Female	Otorhinolaryngology	Medical doctor-specialist	Health center manager
11	26	Male	Virology	PhD	Deputy of development and Director of graduate studies
12	28	Male	Physiotherapy	PhD	Health clinic center manager
13	21	Male	Cardiology	Medical doctor-specialist	Hospital CEO
4	30	Male	Medical and health services management	PhD	Director general of health insurance
15	28	Male	General practitioner	Medical doctor	Hospital CEO

On average, each interview lasted 60–90 min and the interviewees invited for extra meeting if needed. The content of each interview was transcribed verbatim and read several times to find the general content of the participants’ speech. Then, the content entered into the data management software, MAXQDA ver. 10, to identify and label the meaning units represented the main elements and sub-elements of PARIHS frameworks. Eventually, the coded segments categorized under the pre-determined categories, i.e. evidence, context and facilitation.

To confirm the rigor of the study, the extracted data shared with the participants in a two-hour focused group discussion. The participants were asked to identify the position of Iran’s healthcare management on the continuum of evidence, context and facilitation based on the PARIHS framework and rate it as weak or strong (Table 1).

## Results

Analysis of the qualitative data showed that, in terms of evidence, most managers used the local information, service providers and recipients’ experience and local information as a main source of knowledge for decision making and managing the organization. In terms of context, most of the codes were relate to the sub-element of evaluation. Among the facilitators, most managers used others as facilitators.

### Evidence

Analysis of the data in terms of evidence and its sub-element, i.e. research, experience of service providers, experience of service recipients and local information, reported below.

### Research

Some participants used related websites to obtain information.

"At least once or twice a week, I visit the WHO website to check the latest findings on my subject of interest."

They did not often use databases as a source of research findings for decision-making:

"It’s the last thing I would do if I wanted to improve my knowledge about a subject."

Experiences of service providers **(**clinical experience**):** Most managers were using their own and their colleagues’ experiences:

"One of my tasks was to call successful individuals and ask about their viewpoints."

However, most of them were using the comments that took in meetings and councils:

"I created a small group, called the scientific and executive committee, and used the comments of experts in the fields of environmental health and occupational health and diseases."

Results of the councils usually considered for decision-making:

"In weekly meetings, we discussed issues and, then, prepared them for decision making by the university executives."

Some of the managers used the experiences they had gained elsewhere:

"One of the tasks is to empirically use other places as a template; for example, I used some of the works that were beneficial at other universities."

### Comments of service recipients (patient preferences)

The participants in our study rarely took into account service recipients’ viewpoints for decision-making and did not have a regular planning on this matter:

"Getting feedback and comments from service recipients was not systematic, but because I am usually in the workplace environment, I ask for comments and decide accordingly."

This approach also followed by another participant:

"For example, when we have a problem in our hospital concerning payment of salaries, I ask my trusted advisors before decision making."

### Organizational/local information

In the construct of evidence, most of the codes belonged to the sub-element of local information, most of which included the regulations, council minutes and upstream documents.

"Upstream documents itself cannot play a role in the executive field, but can guide the executive system through goal setting."

However, some of the participants considered the instructions as obstacles to their maneuverability: “There are so many regulations and instructions in the ministry of health that have made it impossible to form the structure that a manager has to have on the mind in order to get things done.”

Based on the findings, the level of applying the system data and care provider experiences as a source of evidence in health care managers’ decision making processes rated as high and conversely the level of research findings and service recipients’ experiences utilization rated as low (Table 3).

**Table 3:** Status of knowledge implementation healthcare management in Iran

Core elements	Sub-elements	Rating	
		Low	high
**Evidence**	Research	✓	
	Clinical experience		✓
	patient preferences	✓	
	Local information		✓
**Context**	Culture	✓	
	Leadership	✓	
	Evaluation		✓
**Facilitation**	Skills and attributes	✓	
	Role and purpose	✓	

### Context

Analysis of the data in terms of context and its sub-element, i.e. culture, leadership and evaluation reported below.

### Culture

Most of the participants believed that communicating with others has a key role in the organization. One of the participants regarded communication as a motivating factor:

"I have to hear and know about problems of a nurse and his/her motivation; meeting with a manager is pleasing to some of them and motivates them."

The participants also believed in the system of encouragement and some of them used qualitative tools that designed with the staff’s consent:

"In insurance, payments are based on ratings that employees receive and any creativity or capability is considered when assessing their work."

Some believed in defining organizational culture based on maintaining values and beliefs and were pleased to provide services, but this feeling was fading over time since individuals were paying more attention to personal gain:

"Healthcare is the holiest area of activity because its main concern is humans’ well-being and health, and the look of satisfaction of a patient after receiving the treatment is a joyful experience."

Nevertheless, some of the participants believed that the health system lacked coherence, integrity and strategic objectives:

"In this field, sometimes, decisions are made individually and without harmony; for instance, a plan is instructed at the deputy of health level, but not at the ministerial level, which requires modifications for implementation at the workplace."

### Leadership

A number of participants expressed that clarifying the role of staff was an effective factor in organizational progress:

"Designing a task-based framework in line with the objectives of the organization can help us become well-organized."

However, some of them believed that lack of structure, efficiency and flexibility of the organization prevents the creation of a suitable platform for leadership in the organization:

"One day, we had a university with a limited number of students and faculty members. But after 10 years, the number of faculty members has been doubled; our students have quadrupled and diversity of courses has increased. Nevertheless, nothing has changed in our organizational structure, which forces utilization of employees against the law."

#### Evaluation

According to most participants, evaluation is a major component of the context core element, which is at a high level in the current health system. The participants claimed that they used various methods and multiple resources to evaluate employees:

"We have a triangular model; electronic monitoring, in person and in the system, which utilizes a checklist to determine whether the employee really performed a task or not."

Moreover, the results indicated that feedbacks are often individual and non-systematic:

"I myself send results of the evaluations to colleagues and managers at different levels to check the issues with their employees."

Rating the status of context sub- elements indicated a high level of application of evaluation among healthcare managers as an effective elements to prepare the context for implementing knowledge (high level), while attention to other sub-elements i.e. culture and leadership rated as low level (Table 3).

### Facilitation

The study findings about the facilitation, the third main element of implementation process, presented below in terms of skills, attributes, purpose, and roles of facilitators.

### Skills and attributes

The majority of the participants used consultation, partnership and lobbying to remove obstacles, which are not formal, except in the case of Health Charity Assembly.

"For example, there was a problem with a hospital project. We used consultants in the field of contracting and, even sometimes, asked for the help of technical engineers at Ministry of Intelligence."

### Role and purpose

In this sub-element, great attention paid to occasional contacts with people outside the organization for facilitation, which was not teamwork:

"The health system-related works are based on the bargaining and lobbying power. Hence, we use this solution when facing problems."

The participants also benefited more from the technical facilitation capabilities of politics, such as the Parliament members, provincial officials and headquarters of the ministry:

"For example, we had a problem on building a hospital; we negotiated with the Parliament and the Plan and Budget Organization to get the job done."

Using the PARHIS framework to determine the level of facilitation in health care management showed that all sub-elements of facilitation rated as low (Table 3).

## Discussion

The healthcare system of Iran often manage by the specialized or general physicians. In some cases, however, at the executive level, general practitioners are responsible for administering hospitals. Since they are most likely to manage the healthcare, system based on their experiences and participation in short management courses and workshops on management. Therefore, considering the above conditions and the viewpoints of the participants in this study, the status of knowledge utilization in the health system is not favorable, and the managers mainly run the system based on their specialty and experience as well as relying on medical specialty. As our results show.

Our results indicated the considerable emphasis on context and less attention to evidence and facilitation. This finding is in line with findings of Janson and Forsberg [26]. According to Ward et al., facilitation and context are the most influential factors in decision making [27]. The utilization of knowledge requires the availability of the best evidence, a correct understanding of the structure and goals, culture of change and utilization of effective strategies [28].

It seems that managers pay great attention to the context due to the lack of resources and pay little attention to facilitation because of the inaccurate identification of obstacles and lack of structure and processes in the organization. In addition, managers rarely use research findings, mainly because of the heavy workload and lack of access to exploitable results. Gagnon and Bergeron reported that despite the interest in evidence-based decision making, individuals and organizations create some obstacles in this regard [29]. This issue is not just limited to managers and nurses are also not familiar with evidence-based performance and do not implement research findings in practice [30]. Despite all the efforts, unfortunately, the utilization of knowledge has not yet been institutionalized in Iran’s health system [31]. In other studies, lack of sufficient time has been regarded as a barrier to the implementation of research results [32, 33] and some managers claim that not enough research findings are available in areas that are important to them [34]. Moreover, policymakers generally rely on information other than research findings such as recommendations and routine measurements for decision making [18]. Knowledge utilization is a nonlinear process that begins with needs assessment, situational assessment and needs-based knowledge production, and continues with the evaluation of knowledge transferred to policymakers, peers and public users as well as monitoring and providing feedback [35]. Therefore, it seems that factors such as uncertainty, lack of consensus on research findings and unavailability of brief results for routine decision making by managers may affect this process.

In order to utilize knowledge, all three elements of the PARIHS model (evidence, context and facilitation) must be available [27]. Our findings indicate that the health system of Iran focused mainly on the subject of organizational culture and evaluation, and managers believe that the current culture of the health system lacks coherence. Meanwhile, it is believed that organizational integrity and establishment of coordination between experts and policymakers is essential for implementing changes [18, 36] and encouraging teamwork spirit [37, 38] in the organization. Senge suggested that successful implementation of theories in practice could be only achieved by establishing a collaborative culture via education [39]. It seems that, the lack of organizational integrity and poor team working are the result of dominant culture of the society, prioritizing the personal gain over the organizational success. Furthermore, organizational processes are incoherent and predominately based on individual taste and bargaining. This highlights the need for a reformation in the current management model and comprehensive planning to help build organizational structures based on the actual needs of the health system.

Managers also believe that they should define values and beliefs for employees. In this regard, Ward stated that personnel rating should be carried out in a coherent manner, so that employees become involved in the change process and prioritize the interests of the organization over their own [27]. Other studies have also demonstrated that the interest of individuals, valuing the goals of the organization, belief in change and paying attention to inter-disciplinary activities are effective factors in implementing changes in an organization [40, 41]. Terminating employees who are effective in the implementation of change is dangerous for the organization and these individuals should be encouraged and rewarded [42].

It seems that paying attention to values and beliefs influenced by the dominant culture of the society, but healthcare managers ought to apply a structured encouragement and punishment system with objective indices for the employees. Moreover, designing and implementing a performance-based paying system can motivate employees and improve their efficiency. Our results indicated that managers generally put emphasis on evaluation and utilize multiple methods and resources for this purpose. Other studies have also elucidated that evaluation is a complex, but necessary, component of the environments that seek to implement changes [27, 36, 40, 43].

In Iran, health managers seem to be interested in non-systematic monitoring, use the results as a basis for judgment and fail to make corrective actions. Nevertheless, in recent years, the health system has sought to resolve these issues by using the operational plan monitoring system and performing accreditation.

Our results demonstrated that the element of facilitation has received less attention and the individual skills inside and outside the organization have emphasized more. Some researchers believe that facilitation should be carried out by doing work by others and enabling others [44], while others suggest these two methods of facilitation are mostly carried out in one group [38]. According to Harvey et al., in order to facilitate the change process, first, an interface should be created between the internal staff and external facilitators, which requires specific infrastructure and planning [45]. In addition, access to resources can facilitate knowledge utilization [46]. Thus, for proper facilitation, it is crucial to create a suitable context and allocate resources to this issue [47]. However, it is thought that if clinical managers play their leadership role as internal facilitators, they can successfully implement changes in the organization despite the influence of complex and, sometimes, contradictory elements and environmental fluctuations [48].

It seems that insufficient understanding of the existing problems and lack of a systematic structure have led to the use of lobbying for facilitation, which can be resolved by creating a systematic structure and process.

## Conclusion

Based on the result. It seems necessary to develop a structure in healthcare system for easy and applicable access to research findings, experiences of colleagues and information. Moreover, we need to train managers to accept of the role of insider or outsider facilitators of the organization in healthcare system.

## Data Availability

Since the present study is a qualitative study based on the statements of the participants as pointed out in Section 4 of the informed consent form, the participants only allowed the researchers to publish the findings as a whole. Therefore, we do not have permission to provide research data and materials in public or in an appendix. However, the research team is committed to providing personal data (IPDs) if requested by other researchers by email to the responsible author.

## Abbreviations

MAXQDA: Qualitative & mixed methods data analysis tool
PARIHS: Promoting action on research implementation in health services
W.H.O: World health organization
